# HIV incidence estimates by sex and age group in the population aged 15 years or over, Brazil, 1986-2018

**DOI:** 10.1590/0037-8682-0231-2021

**Published:** 2022-01-28

**Authors:** Célia Landmann Szwarcwald, Paulo Roberto Borges de Souza, Ana Roberta Pati Pascom, Ronaldo de Almeida Coelho, Rachel Abrahão Ribeiro, Giseli Nogueira Damacena, Deborah Carvalho Malta, Maria Cristina Pimenta, Gerson Fernandes Mendes Pereira

**Affiliations:** 1 Fundação Oswaldo Cruz, Instituto de Comunicação e Informação Científica e Tecnológica em Saúde, Rio de Janeiro, RJ, Brasil.; 2 Ministério da Saúde, Departamento de Condições Crônicas e Infecções Sexualmente Transmissíveis, Brasília, DF, Brasil.; 3 Universidade Federal de Minas Gerais, Escola de Enfermagem, Belo Horizonte, MG, Brasil.

**Keywords:** HIV incidence, Estimation, Time trends, Young adults, GBD, Brazil

## Abstract

**INTRODUCTION:**

HIV incidence estimates are essential to monitor the progress of prevention and control interventions.

**METHODS:**

Data collected by Brazilian surveillance systems were used to derive HIV incidence estimates by age group (15-24; 25+) and sex from 1986 to 2018. This study used a back-calculation method based on the first CD4 count among treatment-naïve cases. Incidence estimates for the population aged 15 years or over were compared to Global Burden of Disease Study (GBD) estimates from 2000 to 2018.

**RESULTS:**

Among young men (15-24 years), HIV incidence increased from 6,400 (95% CI: 4,900-8,400), in 2000, to 12,800 (95% CI: 10,800-15,900), in 2015, reaching incidence rates higher than 70/100,000 inhabitants and an annual growth rate of 3.7%. Among young women, HIV incidence decreased from 5,000 (95% CI: 4,200-6,100) to 3,200 (95% CI: 3,000-3,700). Men aged ≥25 years and both female groups showed significant annual decreases in incidence rates from 2000 to 2018. In 2018, the estimated number of new infections was 48,500 (95% CI: 45300-57500), 34,800 (95% CI: 32800-41500) men, 13,600 (95% CI: 12,500-16,000) women. Improvements in the time from infection to diagnosis and in the proportion of cases receiving antiretroviral therapy immediately after diagnosis were found for all groups. Comparison with GBD estimates shows similar rates for men with overlapping confidence intervals. Among women, differences are higher mainly in more recent years.

**CONCLUSIONS:**

The results indicate that efforts to control the HIV epidemic are having an impact. However, there is an urgent need to address the vulnerability of young men.

## INTRODUCTION

Over the past two decades, thanks to the effectiveness of antiretroviral therapy (ART), enormous progress has been made in improving the health and survival of HIV-infected individuals. The success of ART, combined with the benefits of treatment as prevention (TasP) and pre-exposure prophylaxis (PrEP), have generated new expectations surrounding the end of the HIV epidemic^1^.

To ensure early HIV diagnosis and the effective implementation of prompt access to treatment, it is essential to have HIV incidence estimates to describe the current dynamics of the epidemic^2^. Particularly, if estimates can be derived by geographic areas, demographic groups, and risk categories^3^, trends in HIV incidence indicate not only the degree to which HIV transmission is controlled^4^, but also which groups are most at risk for HIV infection in the general population^5^ ^,^ ^6^. Despite the relevant role of HIV incidence in surveillance, estimating the annual number of new infections continues to be challenging in many countries^7^ ^-^ ^9^. 

Historically, the calculation of HIV incidence has been based on reported AIDS cases, using back-calculation models of AIDS incidence, with the assumption that temporal trends in AIDS incidence reflect past trends in HIV incidence^10^. However, the expansion of antiretroviral therapy has lengthened the time to the onset of AIDS^11^, making inferences about HIV incidence based on AIDS reported cases limited. 

HIV incidence can also be calculated based on the change in HIV prevalence estimated at two points in time^12^. The assumption underlying this methodology is that the number of new infections is equal to the number of prevalent cases at the second point in time, minus the number of cases who survived between the two time points. This is the basis of the UNAIDS methodology to estimate key HIV indicators, such as the number of PLHIV, new infections, and AIDS deaths using complex mathematical models^13^. 

In the late 1990s, laboratory tests were developed to estimate HIV incidence in cross-sectional studies^14^. The algorithms are based on laboratory assays that distinguish recent from long-term infections. As in cross-sectional surveys, the main advantage of this type of approach is the use of a single blood sample collected at one point in time, which does not require follow-up on subjects, as would be required in cohort studies^5^. Recent infection testing algorithms (RITAs), incorporating clinical information into the HIV recency assay, have proven to accurately classify recent infections and have been widely used to estimate the incidence of HIV in several countries and different epidemiological settings^15^ ^-^ ^18^, including HIV incidence estimation in two Brazilian cities^19^. However, the logistics of identifying recently acquired infections in routine testing settings may be complicated and costly for continental countries with concentrated HIV epidemics. Moreover, as there is no estimate of cumulative incidence, the biomarker method cannot be used to estimate HIV prevalence and the proportion of undiagnosed infections^3^.

Lately, methods based on the first CD4 count after HIV diagnosis have been developed to estimate HIV incidence in some countries^3^ ^,^ ^6^ ^,^ ^20^ ^-^ ^25^. The main assumption of these models is that among ART treatment-naïve individuals, CD4 cell counts decrease over time^26^. Although it is well-known that a small proportion of people living with HIV (PLHIV) experiences the preservation of CD4^27^, these approaches have the advantage of using a CD4 count back-calculation model to estimate HIV incidence with routinely available data. 

Young people are considered a key population for HIV prevention interventions in Brazil and worldwide^28^. Several risk factors for sexually transmitted infections (STIs) are raised amongst young people, such as being in the beginning of their sexual life, experimenting with high-risk behaviors, and feeling invulnerable^29^. From 2007-2019, reported HIV cases in Brazil rose by 64.6% among young men^30^. Given this epidemiological pattern, the estimation of HIV incidence among Brazilian youth seems timely. 

The present study used data collected by Brazilian surveillance systems to derive HIV incidence estimates among men and women aged 15-24 years from 1986 to 2018. Time trends of HIV incidence rates are compared to those obtained for older age groups. In addition, HIV incidence estimates from 2000 to 2018 were compared to the Global Burden of Diseases (GBD) estimates for males and females aged 15 years or over.

## METHODS

In Brazil, AIDS started to be considered a compulsory notification disease in 1986. The National Information System of Notified AIDS cases (SINAN-AIDS, in Portuguese) was implemented in 1993 and has brought about important advances in terms of systematizing epidemiological data. Since 2014, the notification of HIV cases has also become mandatory, although some states had been reporting HIV cases long before this date.

In the 2000s, in addition to SINAN-AIDS, the surveillance of HIV cases started to rely on other information systems from the Ministry of Health: Mortality Information System (SIM, in Portuguese); Laboratory Examination Control System (SISCEL, in Portuguese); Logistics Control System for Medicines (SICLOM, in Portuguese); and the National System of Notified Tuberculosis cases (SINAN-TB, in Portuguese). All of these databases are linked in order to compose the integrated HIV surveillance information system (SIIHIV, in Portuguese). The integrated information system is used not only to correct the underreporting of cases in SINAN, but also for the clinical monitoring of patients.

In 2013, a model for estimating HIV incidence was proposed based on the first CD4 count among treatment-naïve patients and was applied to SISCEL/SICLOM data to monitor the number of recent infections among men and women from 2004 to 2013^22^ ^,^ ^25^. The approach is based on the first CD4 count after diagnosis among all treatment-naïve HIV infected cases informed to SISCEL to estimate the time since infection. To use the CD4 depletion model in Brazil, the model originally proposed by Lodi et al.^26^ was adapted and specific decline rates by sex and age group were estimated from Brazilian SISCEL data^25^.

The present study proposes an adaptation of the previously developed back-calculation method based on the first CD4 count among treatment-naïve cases^25^ to derive HIV incidence estimates by age-group (15-24; 25 or older) and sex using information from the integrated HIV information system (SIIHIV) from 1986 to 2018. This study was based on the Project “Estimação da incidência de HIV no Brasil utilizando sistemas de informações em saúde” (“Estimation of HIV incidence in Brazil, using health information systems”), which was approved by the Ethics Committee of the Oswaldo Cruz Foundation, Ministry of Health, Brazil (Protocol 2.134.753).

For the analysis, the first HIV detection date among all databases that compose the integrated system was assumed to be the date of HIV diagnosis. In the first step, for each treatment-naïve case of HIV infection informed to SISCEL, the Brazilian CD4 depletion model was used to estimate the time between infection and the first CD4 count, and the time between HIV infection and diagnosis. In the next step, for all cases not informed to SISCEL, the time between HIV infection and diagnosis was estimated by using a multiple imputation procedure using the information source of the first detection date and the notification criterion in SINAN (AIDS or HIV) as predictor variables. 

In similarity to the previous proposal^25^, HIV incidence is calculated as the upper limiting value of the cumulative sum of individuals reported to SISCEL in the same year of HIV infection, in the year following the infection, two years after the infection, and so on. Under the assumption that the probability of being diagnosed less than x years after infection is expressed by a logistic probability distribution (equation 1), HIV incidence is calculated as the upper limit of the cumulative number of cases *N*_*x*_ (equation 2):

$$F(x;\mu ;\sigma )=1/(1+exp(-\pi /\sqrt{3}\cdot z))\text{for}z=(x-\mu )/\sigma$$ (1)

,where µ and σ are the mean and standard deviation of time from infection to diagnosis and *x* is the number of years after infection. 

$N_{x}=I.F(x;\mu ;\sigma )$ , where I is HIV incidence. (2)

To estimate μ, σ, and HIV incidence (*I*), an iterative procedure was used with an initial guess for σ to generate successive approximations to a solution. The detailed description of the estimation method of the parameters μ and σ can be found in a previous publication^25^ (Supplementary methodology section). 

To estimate HIV incidence by sex and age group, age at infection was estimated as age at diagnosis minus the time between infection and diagnosis. Because HIV incidence is estimated for people aged 15 or over, if the date of infection is estimated before age 15, it is set to the date the person reaches 15 years of age^3^. Using this approach, HIV incidence was estimated by sex and age group (15-24; 25+) in Brazil from 2000 to 2013 and the proportion of cases reported within the first year of HIV infection (*p*_*1*_ ). 

As the estimated HIV incidence loses accuracy with a small number of observations^25^, the number of HIV cases diagnosed within the first year of infection was used to generate estimates in recent years. To estimate HIV incidence from 2014 to 2018, we first predicted the proportion of cases reported within the first year of HIV infection by fitting a regression model to the logit(*p*_*1t*_ ) varying with time (t) (t=0,…..,13), using t, t^2^, and ln(t) as independent variables and the upper limit of 0.70:

$$Ln\left(\frac{p_{1t}}{\left(0.70-p_{1t}\right)}\right)=a+b_{1}\cdot t+b_{2}\cdot t^{2}+b_{3}\cdot ln(t)$$ (3)

The estimated coefficients of determination (R^2^) were 0.96 for men aged 15-24 years, men ≥25 years old, women aged 15-24 years, and 0.92 for women >=25 years. 

The estimate of HIV incidence from 2014 to 2018 was then given by the ratio of the number of newly infected individuals diagnosed within the first year of HIV infection and the predicted proportion $\left({\hat{p}}_{1t}\right)$ . Confidence intervals for HIV incidence were based on 95% confidence intervals (95% CI) for the predicted proportions. 

Incidence estimates in the Brazilian population aged 15 years or over were calculated by summing up incidence estimates by age group. Information on population by age group and sex were used to estimate HIV incidence rates. HIV incidence estimates for males and females aged 15 years or older were compared to the Global Burden of Diseases (GBD) estimates.

The software Statistical Package for the Social Sciences (IBM SPSS for Windows), version 22.0, was used for the statistical analysis.

The GBD methodology used to estimate HIV incidence in Brazil is based on cause-specific vital registration information. Mortality data were analyzed at the subnational level and were corrected for garbage coding and HIV misclassification. HIV incidence estimates were generated by back-calculation from mortality data, using assumptions of disease progression and survival. For the cohort incidence bias adjustment, a process was developed by GBD driven by Spectrum mortality and incidence cohort survival estimates. The detailed methodology is described in Murray et al. (2019)^31^.

## RESULTS

Estimates of HIV incidence by sex and age group from 2000 to 2018 are presented in Table 1. Among young men (15-24 years), results show HIV incidence increased from 6,400 (95% CI: 4,900-8,400) in 2000 to 12,800 (95% CI: 10,800-15,900), in 2015, and, after that year, began to decrease slowly. Among young women (15-24 years), results show HIV incidence decreased from 5,000 (95% CI: 4,200-6,100), in 2000, to 3,200 (95% CI: 3,000-3,700), in 2018. In this age group, the male-female incidence ratio was 1.3 in 2000 and increased to 3.7 in 2018. Among men aged 15-24 years, HIV incidence rates reached values higher than 70 per 100,000 inhabitants from 2014 to 2016. Among women of the same age group, in 2018, the incidence rate was 18.6 (95% CI:16.7-22.1) per 100,000, almost four times smaller than among men.

In the age group of 25 years or over, men showed an increasing trend until 2015 but at a much slower pace than in the youngest age group, from 16,600 (95% CI: 14,600-19,200) to 24,300 (95% CI: 22,700-26,500), in 2015, showing a slight decrease after this year. HIV incidence among women aged 25 years or over showed little variation in the period, with approximately 11,500 new infections per year. In the oldest age group, the male-female incidence ratio increased from 1.6 to 2.2, from 2000 to 2018. Comparing HIV incidence rates, the youngest men show the highest rates of all groups (Table 1). 

**TABLE 1:** HIV incidence and HIV incidence rates (per 100,000 inhabitants) by sex, age group, and year of HIV infection. Brazil, 2004-18.

Year	Males											
	15-24 years						25 years or over					
	Incidence (x1000)	95% CI		Rate	95% CI		Incidence	95% CI		Rate	95% CI	
		LL	UL	(/100000)	LL	UL	(x1000)	LL	UL	(/100000)	LL	UL
2000	6.4	4.9	8.4	37.7	28.8	50.0	16.6	14.6	19.2	41.3	36.1	47.7
2001	6.7	5.3	8.4	39.4	31.5	49.8	17.4	15.6	19.5	42.0	37.7	47.1
2002	7.1	5.7	8.9	41.7	33.4	52.7	17.8	16.0	20.0	42.0	37.8	47.1
2003	7.5	6.0	9.4	44.0	35.4	55.2	18.3	16.5	20.5	42.1	37.9	47.1
2004	7.8	6.3	9.8	45.8	37.2	57.1	18.5	16.7	20.6	41.5	37.5	46.3
2005	8.0	6.5	9.9	46.7	38.0	57.9	18.7	16.9	20.9	41.0	37.1	45.6
2006	8.1	6.6	10.0	47.1	38.4	58.4	19.1	17.3	21.2	40.7	36.9	45.3
2007	8.2	6.7	10.2	47.7	39.1	59.0	19.5	17.7	21.7	40.6	36.9	45.1
2008	8.4	6.9	10.3	48.6	39.9	59.8	19.9	18.1	22.0	40.4	36.7	44.7
2009	8.6	7.2	10.6	49.7	41.2	60.8	20.2	18.4	22.2	39.9	36.5	43.9
2010	9.1	7.6	11.1	52.5	43.9	63.6	20.5	18.8	22.4	39.5	36.3	43.2
2011	9.9	8.3	11.8	56.5	47.8	67.8	20.8	19.2	22.6	39.1	36.2	42.5
2012	10.6	9.0	12.7	60.6	51.6	72.5	21.3	19.8	23.0	39.0	36.3	42.2
2013	11.6	9.8	13.9	65.8	56.0	79.0	22.4	20.9	24.2	40.1	37.4	43.4
2014	12.4	10.5	15.2	70.6	59.8	86.1	23.7	22.2	25.8	41.4	38.7	44.9
2015	12.8	10.8	15.9	72.5	61.1	90.2	24.3	22.7	26.5	41.3	38.6	45.0
2016	12.6	10.7	16.1	71.3	60.2	90.6	24.0	22.5	26.2	39.8	37.3	43.5
2017	12.0	10.2	15.5	67.5	57.6	87.2	23.2	21.8	25.3	37.4	35.3	40.9
2018	11.9	10.9	16.4	66.5	61.2	91.8	23.0	21.9	25.1	36.2	34.5	39.5
Year	**Females**											
	**15-24 years**						**25 years or over**					
	**Incidence (x1000)**	95% CI		**Rate**	**95% CI**		**Incidence**	**95% CI**		**Rate**	**95% CI**	
		**LL**	**UL**	**(/100000)**			**(x1000)**	**LL**	**UL**	**(/100000)**	**LL**	**UL**
2000	5.0	4.2	6.1	29.6	24.9	35.8	10.7	9.0	13.0	24.6	20.7	30.1
2001	5.1	4.5	5.9	30.3	26.6	34.9	11.2	9.8	13.1	25.3	22.1	29.5
2002	5.1	4.5	5.8	29.8	26.4	34.2	11.7	10.3	13.6	25.7	22.6	29.8
2003	5.1	4.5	5.8	29.9	26.6	33.9	12.0	10.6	13.9	25.7	22.7	29.7
2004	5.0	4.4	5.6	29.1	26.1	32.9	12.1	10.7	13.9	25.2	22.3	28.9
2005	4.8	4.4	5.4	28.4	25.6	31.9	12.1	10.7	13.8	24.5	21.8	28.0
2006	4.6	4.2	5.2	27.3	24.6	30.5	11.9	10.6	13.6	23.6	21.0	26.9
2007	4.5	4.1	5.0	26.4	23.9	29.5	11.8	10.6	13.5	22.8	20.4	26.0
2008	4.4	4.0	4.9	25.6	23.3	28.5	11.8	10.6	13.4	22.2	19.9	25.2
2009	4.2	3.9	4.7	24.7	22.6	27.4	11.8	10.6	13.3	21.6	19.4	24.3
2010	4.1	3.8	4.5	23.9	22.0	26.3	11.7	10.6	13.1	20.9	19.0	23.4
2011	4.0	3.7	4.4	23.3	21.6	25.5	11.5	10.5	12.8	20.1	18.3	22.3
2012	3.9	3.7	4.3	23.0	21.3	25.0	11.4	10.5	12.7	19.4	17.8	21.5
2013	3.9	3.6	4.2	22.6	21.0	24.7	11.5	10.6	12.8	19.1	17.5	21.2
2014	3.9	3.6	4.3	22.5	20.9	24.8	11.6	10.7	13.0	18.8	17.2	21.0
2015	3.8	3.5	4.2	22.0	20.3	24.4	11.5	10.5	13.1	18.1	16.5	20.6
2016	3.6	3.3	4.0	20.6	18.9	23.1	11.0	10.0	12.7	16.9	15.4	19.4
2017	3.4	3.1	3.8	19.5	17.9	22.2	10.6	9.7	12.4	15.8	14.5	18.5
2018	3.2	3.0	3.7	18.6	17.2	21.4	10.4	9.6	12.3	15.1	13.9	17.9

**FIGURE 1:**
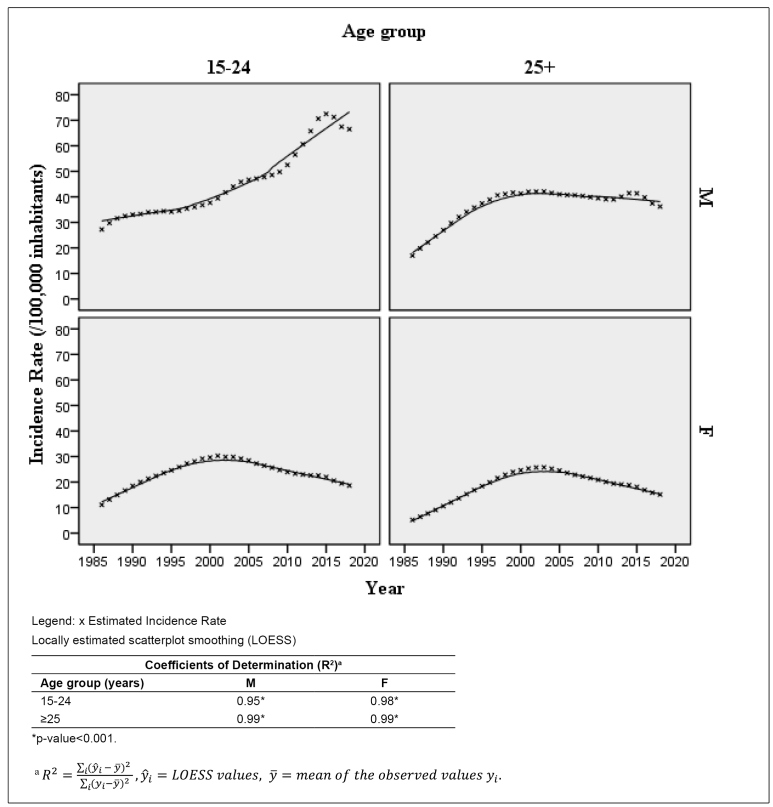
HIV Incidence Rates (per 100,000 inhabitants) by sex and age group. Brazil. 1986-2018.

Trends of HIV incidence rates by age group and sex can be examined in Figure 1. Among the youngest males, after years of pronounced growth, there is a small decline in HIV incidence rates after 2016. Among men aged 25 or over, the growth trend at the beginning of the epidemic changes to a stable level after the year 2000. Both female groups show the same pattern, a rising trend from 1986 to 2005 and a decreasing trend from 2006 to 2018, although the decrease among the youngest age group is sharper.

In 2018, the estimated number of new infections in Brazil was 48,500 (95% CI: 45300-57500), 34,800 (95% CI: 32800-41500) men, representing 72% of the total, and 13,600 (95% CI: 12,500-16,000) women. For the total population aged 15 years or over, the HIV incidence rate was 29.0 per 100,000 (95% CI: 27.1-34.3), in 2018, with 42.9 (95% CI: 40.3-51.0) among men and 15.8 (95% CI: 14.6-18.6) among women. Among males, although a small but significant annual decreasing rate was found in the period 2000-2018, peak incidence rates were observed in 2014, 2015, and 2016, while for females, a continuous decrease was found after 2003 (Table 2). 

Comparison with GBD estimates shows close values for men aged 15 years or over, with all confidence intervals overlapping. Small differences were found in recent years: the average ratio between the estimates was 1.06 but reaches 1.16 in 2018. Among women, similar estimates were also found until 2009. However, GBD estimates show an upward trend from 2009 to 2014 and steadiness after year 2014, with approximately 22,000 new infections each year, while Brazilian estimates present a decreasing trend so that the differences widen at the end of the time series. Among women, the average ratio between estimates was 1.05 until 2009 but attains values higher than 1.50 in the last three years (Table 2).

**TABLE 2:** Comparison of HIV incidence and HIV incidence rate estimates (per 100,000 inhabitants) with GBD estimates by sex and year of HIV infection. Brazil, 2000-2018.

Year	Males											
	Brazilian estimates						GBD estimates					
	Incidence (x1000)	95% CI		Rate	95% CI		Incidence	95% CI		Rate	95% CI	
		LL	UL	(/100000)	LL	UL	(x1000)	LL	UL	(/100000)	LL	UL
2000	23.0	19.4	27.7	40.2	34.0	48.4	27.9	21.4	34.0	48.8	37.4	59.5
2001	24.0	20.9	27.9	41.2	35.9	47.9	25.3	20.3	30.6	43.4	34.8	52.5
2002	24.9	21.7	28.9	41.9	36.5	48.7	24.6	19.7	29.8	41.4	33.2	50.2
2003	25.8	22.5	29.9	42.6	37.2	49.4	24.7	19.6	29.8	40.8	32.4	49.3
2004	26.3	23.1	30.4	42.7	37.4	49.3	24.9	20.0	30.0	40.4	32.4	48.6
2005	26.7	23.5	30.8	42.5	37.3	49.0	25.5	20.7	30.4	40.6	32.9	48.4
2006	27.2	23.9	31.3	42.5	37.3	48.8	26.4	21.2	31.4	41.2	33.1	49.0
2007	27.8	24.5	31.9	42.5	37.4	48.8	27.3	21.9	32.5	41.8	33.5	49.8
2008	28.3	25.0	32.4	42.5	37.6	48.6	28.5	22.8	33.9	42.8	34.2	50.9
2009	28.8	25.6	32.8	42.4	37.7	48.3	30.3	23.6	36.4	44.6	34.7	53.6
2010	29.6	26.5	33.5	42.7	38.2	48.3	32.3	24.8	38.8	46.6	35.8	56.0
2011	30.6	27.6	34.4	43.4	39.0	48.8	34.0	25.8	41.1	48.1	36.5	58.2
2012	31.9	28.8	35.7	44.2	40.0	49.6	36.1	27.3	43.5	50.1	37.9	60.4
2013	34.0	30.7	38.1	46.2	41.8	51.9	38.2	28.5	46.3	52.0	38.8	63.0
2014	36.2	32.7	40.9	48.3	43.6	54.6	40.1	29.8	48.4	53.5	39.7	64.6
2015	37.1	33.5	42.4	48.5	43.8	55.5	40.4	30.1	48.7	52.8	39.4	63.7
2016	36.6	33.1	42.3	46.9	42.5	54.2	40.4	30.4	48.8	51.8	39.0	62.5
2017	35.2	32.1	40.8	44.2	40.2	51.3	40.4	30.6	49.1	50.7	38.4	61.7
2018	34.8	32.8	41.5	42.9	40.3	51.0	40.4	30.7	49.3	49.7	37.8	60.7
Year	**Females**											
	**Brazilian estimates**						**GBD estimates**					
	**Incidence (x1000)**	95% CI		**Rate**	**95% CI**		**Incidence**	**95% CI**		**Rate**	**95% CI**	
		**LL**	**UL**	**(/100000)**			**(x1000)**	**LL**	**UL**	**(/100000)**	**LL**	**UL**
2000	15.7	13.2	19.1	26.0	21.9	31.7	19.4	14.5	23.6	32.2	24.1	39.2
2001	16.4	14.3	19.0	26.7	23.3	31.0	17.6	13.6	21.5	28.7	22.2	35.0
2002	16.8	14.8	19.4	26.8	23.6	31.0	17.1	12.9	21.2	27.3	20.6	33.9
2003	17.1	15.1	19.6	26.8	23.7	30.8	16.8	12.7	21.2	26.4	19.9	33.3
2004	17.1	15.2	19.5	26.2	23.3	30.0	16.6	12.6	21.0	25.5	19.4	32.3
2005	16.9	15.1	19.2	25.5	22.8	29.0	16.5	12.6	21.0	24.9	19.0	31.7
2006	16.5	14.8	18.8	24.5	21.9	27.8	16.7	12.7	21.2	24.7	18.8	31.4
2007	16.3	14.6	18.5	23.7	21.3	26.9	16.8	12.8	21.5	24.4	18.6	31.2
2008	16.2	14.6	18.3	23.0	20.7	26.0	17.2	13.0	22.2	24.5	18.5	31.6
2009	16.0	14.5	18.0	22.3	20.2	25.1	17.8	13.1	23.3	24.8	18.3	32.5
2010	15.8	14.4	17.6	21.6	19.7	24.1	18.6	13.5	24.6	25.4	18.5	33.7
2011	15.5	14.2	17.2	20.8	19.1	23.1	19.3	13.8	25.3	25.9	18.5	33.9
2012	15.4	14.1	17.0	20.2	18.6	22.3	20.2	13.8	26.1	26.5	18.1	34.3
2013	15.4	14.2	17.0	19.9	18.3	22.0	21.2	14.7	27.2	27.3	18.9	35.0
2014	15.5	14.3	17.3	19.6	18.0	21.8	22.4	15.5	28.4	28.3	19.6	35.8
2015	15.3	14.0	17.3	18.9	17.3	21.4	22.2	15.5	28.0	27.4	19.2	34.6
2016	14.6	13.3	16.7	17.6	16.1	20.2	22.4	15.7	28.1	27.1	19.0	34.0
2017	14.0	12.8	16.2	16.6	15.2	19.2	22.4	15.8	28.0	26.6	18.7	33.2
2018	13.6	12.5	16.0	15.8	14.6	18.6	22.4	15.9	28.0	26.0	18.5	32.5
Year	**Total**											
	**Brazilian estimates**						**GBD estimates**					
	**Incidence (x1000)**	95% CI		**Rate**	**95% CI**		**Incidence**	**95% CI**		**Rate**	**95% CI**	
		**LL**	**UL**	**(/100000)**	**LL**	**UL**	**(x1000)**	**LL**	**UL**	**(/100000)**	**LL**	**UL**
2000	38.7	32.6	46.7	32.9	27.8	39.8	47.3	36.1	57.3	40.3	30.7	48.8
2001	40.4	35.2	46.9	33.8	29.5	39.2	42.9	34.0	52.0	35.9	28.4	43.5
2002	41.7	36.4	48.3	34.2	29.9	39.6	41.7	32.8	50.7	34.2	26.9	41.6
2003	42.9	37.6	49.5	34.5	30.3	39.9	41.5	32.7	50.7	33.4	26.3	40.8
2004	43.4	38.2	49.9	34.3	30.2	39.4	41.5	32.8	50.6	32.8	25.9	40.0
2005	43.6	38.5	50.0	33.8	29.8	38.7	42.0	33.4	51.1	32.5	25.9	39.6
2006	43.7	38.7	50.1	33.2	29.4	38.0	42.9	33.9	52.1	32.6	25.8	39.6
2007	44.1	39.1	50.4	32.9	29.1	37.5	44.2	35.0	53.6	32.9	26.1	39.9
2008	44.5	39.6	50.6	32.5	28.9	37.0	45.8	35.7	55.8	33.5	26.1	40.8
2009	44.8	40.1	50.7	32.1	28.7	36.4	48.0	36.8	59.5	34.4	26.4	42.6
2010	45.4	40.9	51.1	31.9	28.7	35.9	50.9	38.3	63.0	35.8	26.9	44.3
2011	46.2	41.8	51.6	31.8	28.8	35.6	53.3	39.8	65.9	36.7	27.4	45.4
2012	47.2	43.0	52.7	31.9	29.0	35.6	56.2	41.7	69.1	37.9	28.1	46.6
2013	49.4	44.9	55.2	32.7	29.7	36.5	59.3	43.5	72.6	39.2	28.8	48.0
2014	51.7	47.0	58.2	33.5	30.5	37.8	62.5	45.4	76.4	40.5	29.4	49.5
2015	52.5	47.5	59.7	33.3	30.2	37.9	62.6	46.0	76.4	39.8	29.2	48.5
2016	51.2	46.4	58.9	31.9	28.9	36.7	62.8	46.4	76.4	39.1	28.9	47.6
2017	49.1	44.8	57.0	30.0	27.4	34.8	62.9	46.8	77.8	38.4	28.6	47.5
2018	48.5	45.3	57.5	29.0	27.1	34.3	62.8	47.0	77.4	37.5	28.1	46.3

**Information sources:** Brazilian estimates - Department of Chronic Conditions and Sexually Transmitted Diseases (DCCI), Ministry of Health. GBD estimates: https://vizhub.healthdata.org/gbd-compare/.

**FIGURE 2:**
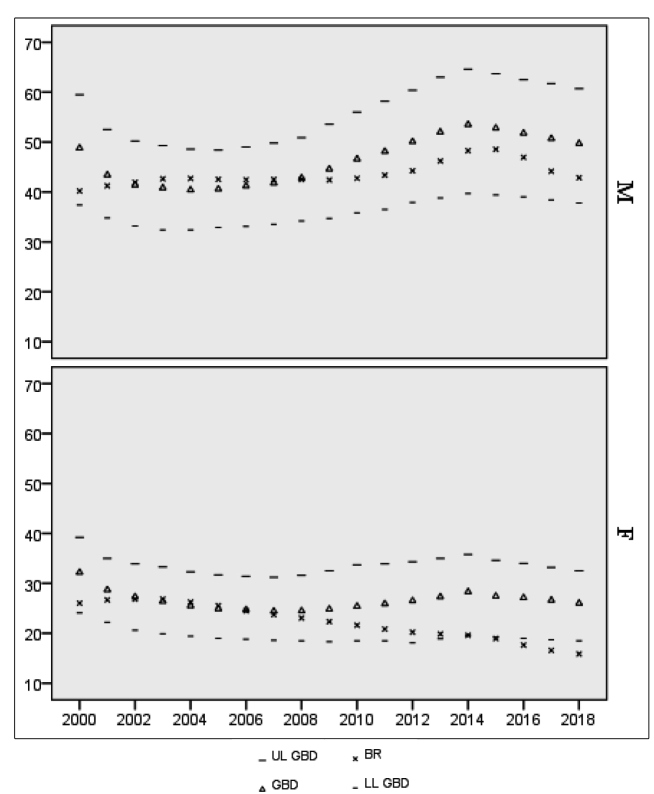
Brazilian and GBD estimates of HIV Incidence Rates (per 100,000 inhabitants) by sex. Brazil. 2000-2018. *GBD - GBD Incidence Rate (/100,000 inhabitants) estimate. UL GBD - 95% CI Upper limit of the GBD estimate. LL GBD - 95% CI Lower limit of the GBD estimate. BR - Brazilian Incidence Rate (/100,000 inhabitants).

In Figure 2, trends of Brazilian and GBD incidence rate estimates are compared. Among men aged 15 years or over, all Brazilian estimates are within the GBD 95% CIs. Time trends are also similar, but a sharper decrease was found in the Brazilian estimates in recent years. Among women, Brazilian rates are within the GBD 95% CIs until 2014. However, as trends in incidence rates diverge after that year, Brazilian estimates move away from the lower limits of the GBD CI. 

Progress indicators of epidemic control interventions are presented in Table 3. Annual growth rates of HIV incidence rates in the period 2000-2018 indicate that the highest annual growth was presented by the youngest age group (3.7%). Men aged 25 years or over showed a small but significant decreasing rate (-0.5% per year). Both female age groups showed decreasing rates of variation (-2.7% and -2.9%), among women aged 15-24 and 25 or over, respectively.

Improvements in the average time from infection to diagnosis were found for all age groups and both sexes (Table 3). Among men aged 15-24 years, the average time decreased from 9.0 to 3.9 years, and the estimated proportion of cases diagnosed less than one year after infection increased from 11.0% to 52.5% from 2000 to 2018. Males aged 25 years or over presented the lowest average time from infection to diagnosis (2.8 years), and the highest proportion of people diagnosed in the same year of infection (60.6%) in 2018. Among the youngest men who became HIV infected from 2000 to 2018, the proportion of undiagnosed cases by 2018 was 22.6%, and among the youngest women, 11.9%, while among the oldest, the proportions were 13.4% among males and 12.9% among females. There is an increase in the proportion of HIV cases with antiretroviral dispensation within the first year of infection, reaching 48.1% among men and 42.6% among women in 2018.

**TABLE 3:** Mean time from HIV infection to diagnosis. Proportion (%) of HIV infected cases diagnosed within the first year of infection. Proportion (%) with ART dispensation within the first year of HIV infection. Proportion of undiagnosed cases from 2010 to 2018. HIV incidence rate annual growth^a^ from 2000 to 2018 by sex and age group. Brazil. selected years.

Sex	Age Group	Year of HIV infection	Mean time (years)	Proportion (%) of diagnosed cases in the HIV infection year	Proportion (%) with ART dispensation in the HIV infection year	Proportion (%) of undiagnosed cases from 2000 to 2018	HIV incidence rate annual variation^a^ (%)
Males	15-24	2000	9.0	11.5	0.5	22.6	3.7*
		2005	8.6	11.6	0.2		
		2010	6.1	20.9	1.0		
		2014	5.0	35.2	14.7		
		2018	3.9	52.5	45.0		
	25+	2000	5.0	26.9	3.8	13.4	-0.5*
		2005	4.3	27.6	1.9		
		2010	3.5	35.2	5.3		
		2014	3.2	50.0	24.4		
		2018	2.8	60.6	50.1		
	Total	2000	6.5	22.7	2.4	16.3	0.7*
		2005	5.6	22.8	1.2		
		2010	4.8	30.7	3.6		
		2014	3.7	44.6	20.6		
		2018	3.4	57.8	48.1		
Females	15-24	2000	5.9	23.8	2.2	11.9	-2.7*
		2005	5.3	31.1	2.4		
		2010	4.6	40.0	8.4		
		2014	4.1	49.0	22.1		
		2018	3.3	56.4	41.9		
	25-+	2000	3.5	28.8	4.2	12.9	-2.9*
		2005	3.3	33.5	2.8		
		2010	3.2	39.2	6.7		
		2014	3.0	50.7	23.1		
		2018	2.9	57.1	43.1		
	Total	2000	5.3	27.2	3.3	12.6	-2.8*
		2005	4.7	32.8	2.6		
		2010	4.1	39.4	7.0		
		2014	3.7	50.2	22.6		
		2018	3.0	56.6	42.6		

*p-value less than 0.01.

^a^ Estimated by an exponential regression model assuming that incidence rates vary from 2000 to 2018 with a constant percent variation by year.

## DISCUSSION

In this study, surveillance available data was used to estimate HIV incidence categorized by sex and age group in Brazil. The approach is applicable at granular levels to all countries that monitor the clinical information of patients (CD4 count and ART). The procedure used in this study represents a simplification when compared to the previous model^25^ and uses the number of HIV cases diagnosed within the first year of infection to generate estimates in recent years.

This method produced estimates consistent with those of the UNAIDS report 2019^32^. Like our results, estimates resulting from an age-structured deterministic model with reporting and treatment rates showed a second wave of infections after 2001 for both sexes, with the female curve decreasing after 2009^33^.

Regarding GBD estimates among men, HIV incidence rates similar, as are time trends from 2000 to 2018. Although all CIs overlap, small differences were found in recent years, as Brazilian incidence rates based on surveillance data has a sharper decline after 2015 than GBD estimates. Among women, similar estimates were also found up to 2009, but different trends were found after this year when Brazilian estimates move away from the lower limit of the GBD CIs.

Results of this study showed an increase in the proportion of people diagnosed within the first year of infection, a decrease in the average time from infection to diagnosis, and improvements in the proportion of cases starting antiretroviral therapy less than one year after HIV infection. The implementation of the strategy of treatment as prevention (TasP) in December 2013, early HIV diagnosis, and universal treatment at federal, state, and municipality levels, and other prevention interventions, such as pre-exposure prophylaxis (PreP), are having an impact on the control of the HIV epidemic in Brazil^34^ ^,^ ^35^. A recent study with patients on ART therapy in Brazil showed an increase in the proportion of cases with viral load suppression^36^. PrEP has also been implemented since December 2017 in Brazil^37^ ^,^ ^38^ and may also be reflected in these results. 

Decreases in undiagnosed HIV infection may well be attributable to intensified testing efforts in Brazil. These include multiple services for HIV testing, mobile health units, self-testing, and community-based rapid HIV testing strategies in partnership with non-governmental organizations (NGOs)^39^ ^,^ ^40^. Various national studies indicate that the proportion of people tested for HIV infection has increased, especially among most-at-risk population groups. Comparison of two rounds of biological behavioral surveillance surveys (BBSS) among men who have sex with men (MSM) showed the proportion of those who have never been tested for HIV decreased from 49.8%, in 2009, to 33.8%, in 2016^41^. Among female sex workers, relevant improvements were also found, with the proportion of HIV testing in the last 12 months increasing from 20.3% to 39.3%^42^. 

The continuous decrease in HIV incidence among young women may be attributed to government policy to reduce vertical transmission in Brazil^43^. Reduction in the number of HIV cases among children under five years of age and the proportion of HIV infected children among those exposed are clear indicators of the impact of the routine antenatal HIV screening and other care practices to prevent mother to child transmission^44^. In contrast, the group of youngest men (15-24 years of age) is the only group with a significant increase in the HIV incidence trend from 2000 to 2018. Furthermore, the highest percentage of undiagnosed incident cases from 2010 to 2018 was also found in this group. 

One limitation of the Brazilian surveillance integrated system is the large proportion of missing data in the risk category, restricting HIV incidence analyses to available variables, such as age group, sex, and area of residence. However, the literature highlights that young men who has sex with men (MSM) have an increased risk of HIV infection, for which HIV infection rates are higher and are rising relative to the general population. Three nationwide studies of military recruits in 2002, 2007, and 2016 reported HIV prevalence among MSM of 0.56%, 1.23%, and 1.32%, respectively, while the overall recruit population prevalence remained stable, and much lower (0.09%, 0.11%, and 0.12%)^28^ ^,^ ^45^. In a study conducted in two Brazilian cities using laboratory tests to distinguish recent from long-term infections, the estimated incidence rate was greater than 1% among MSM, more than 30 times greater than for heterosexual men in both cities^19^. 

Other settings have experienced similar rises in incidence among MSM^46^ ^,^ ^47^. Despite earlier concerns about an increase in heterosexual cases and “feminization” of the epidemics in Brazil, it seems the actual scenario is the predominance of HIV infection among men, especially among the youngest age group. A recent online survey among MSM in Brazil revealed a low proportion of perceived HIV risk (26%). Moreover, the younger age group increased the odds of binge drinking and unprotected receptive anal sex, and lowered odds of perceived HIV risk^48^. The lack of HIV risk perception is of concern because this may undermine control and prevention efforts, such as PreP. It can therefore be concluded that there is an urgent need to address the vulnerability of young MSM and identify interventions that could reshape notions and perceptions of risk in this group. 

Furthermore, the present study indicates the need for expansion of public prevention policies focused on adolescents with more effective communication strategies, including the development of knowledge that involves motivation for a safer behavior^49^. Nevertheless, setbacks of religious and conservative positions have precluded sexual education activities in schools, which may explain the rising rates of unsafe sex among young people and the lack of risk perception, representing major challenges for public health policies aimed at controlling HIV epidemics in Brazil^50^. 

Limitations of the Brazilian approach stem from possible violations of underlying CD4 model assumptions and from the imputation procedure used to estimate time from HIV infection to diagnosis. Additionally, as the estimation process relies on modelling the proportion of cases diagnosed within the first year of infection, estimates are uncertain in the more recent years, where we have less information about the expansion of early diagnosis, especially given the government policies of TasP and incentives to HIV testing. Although these limitations might influence the results, the method generated estimates consistent with that of other studies using different methodologies. 

Updates to these estimates will enable professionals to monitor progress and plan effective interventions. Moreover, depending on the granularity of the data, the model can be used to derive estimates in other sub-populations and focus interventions on the most challenging population groups at subnational levels.
